# Autism, Gastrointestinal Symptoms and Modulation of Gut Microbiota by Nutritional Interventions

**DOI:** 10.3390/nu11112812

**Published:** 2019-11-18

**Authors:** Maria Vittoria Ristori, Andrea Quagliariello, Sofia Reddel, Gianluca Ianiro, Stefano Vicari, Antonio Gasbarrini, Lorenza Putignani

**Affiliations:** 1 Unit of Human Microbiome, Children’s Hospital and Research Institute “Bambino Gesù”, IRCCS, Piazza Sant’Onofrio 4, 00165 Rome, Italy; mvittoria.ristori@opbg.net (M.V.R.); andrea.quagliariello@opbg.net (A.Q.); sofia.reddel@opbg.net (S.R.); 2 Dipartimento di Gastroenterologia, Università Cattolica del Sacro Cuore, Fondazione Policlinico Universitario A. Gemelli IRCCS, Largo A. Gemelli 8, 00168 Rome, Italy; gianluca.ianiro@unicatt.it; 3 Neuropsichiatria dell’infanzia e dell’adolescenza, Children’s Hospital and Research Institute “Bambino Gesù”, IRCCS, Piazza Sant’Onofrio 4, 00165 Rome, Italy; stefano.vicari@opbg.net; 4 Istituto di Patologia Speciale Medica, Università Cattolica del Sacro Cuore, 00168 Rome, Italy; 5 UOC Medicina Interna e Gastroenterologia, Area Gastroenterologia ed Oncologia Medica, Dipartimento di Scienze Gastroenterologiche, Endocrino-Metaboliche e Nefro-Urologiche, Fondazione Policlinico Universitario A. Gemelli IRCCS, 00168 Rome, Italy; 6 Units of Parasitology and Human Microbiome, Children’s Hospital and Research Institute “Bambino Gesù”, IRCCS, Piazza Sant’Onofrio 4, 00165 Rome, Italy

**Keywords:** autism spectrum disorders (ASD), diet, nutritional status, anthropometry, metabolites, gastrointestinal symptoms, gut microbiome

## Abstract

Autism spectrum disorder (ASD) is a complex behavioral syndrome that is characterized by speech and language disorders, intellectual impairment, learning and motor dysfunctions. Several genetic and environmental factors are suspected to affect the ASD phenotype including air pollution, exposure to pesticides, maternal infections, inflammatory conditions, dietary factors or consumption of antibiotics during pregnancy. Many children with ASD shows abnormalities in gastrointestinal (GI) physiology, including increased intestinal permeability, overall microbiota alterations, and gut infection. Moreover, they are “picky eaters” and the existence of specific sensory patterns in ASD patients could represent one of the main aspects in hampering feeding. GI disorders are associated with an altered composition of the gut microbiota. Gut microbiome is able to communicate with brain activities through microbiota-derived signaling molecules, immune mediators, gut hormones as well as vagal and spinal afferent neurons. Since the diet induces changes in the intestinal microbiota and in the production of molecules, such as the SCFA, we wanted to investigate the role that nutritional intervention can have on GI microbiota composition and thus on its influence on behavior, GI symptoms and microbiota composition and report which are the beneficial effect on ASD conditions.

## 1. Introduction

Autism spectrum disorder (ASD) is a complex behavioral syndrome that occurs before the third year of life and which affects several spheres of the normal mental development. Children with ASD are characterized by speech and language disorders, intellectual impairment, learning and motor dysfunctions [1]. The effects and the severity of symptoms of ASD are different in each person, with a wide range of types and severity of behavior. Verbal and nonverbal intelligence quotients (IQs) are highly variable in ASD [2] and Repetitive and Restricted Behaviors (RRBs) can range from low-level stereotyped motor behaviors to higher order behaviors, such as insistence on sameness [1]. Recently, an increase in the diagnosis of ASD has been reported with an average of 1 case for every 88 children [1,3]. It is currently believed that these disorders result from alterations in pre- and/or post-natal neurological development [1]. Indeed, it has been proposed that these complex behavioral features are associated with atypical patterns of functional connectivity (FC), compared with typically developing (TD) individuals [4,5]. These neurodevelopmental abnormalities lead to the child’s impairment in the ability to relate with others in the first years of life, causing dramatic cognitive, affective and behavioral effects, which need to be approached in the family and at school. 

Among the pathogenic factors of ASD there are very strong genetic components, where heritability has been estimated to be from 60% [6,7] to more than 80% [8]. The genetic factors affecting ASD are very heterogeneous [9,10] and there are few genes whose association to ASD have been well characterized [11,12], for example SH3 and multiple ankyrin repeat domain 3 (SHANK3) [11,13,14,15], contactin associated protein-like 2 (CNTNAP2) [16,17], and more recently, chromodomain helicase DNA binding protein 8 (CHD8) [18]. In particular, both de novo mutations and deletions in the SHANK3 gene have been related to autism. Furthermore, Mark E. Obrenovich et al. have shown that metal ion homeostasis is altered in ASD children and involve the deposition of several divalent cations, as demonstrated in a complex autosomal dominant disorder characterized by ASD, which is known as Timothy syndrome [19,20]

Next to genetic factors, the environmental elements that are implicated in the increase of ASD risk seem to include: air pollution, exposure to pesticides, maternal infections, dietary factors, maternal diabetes, stress, medications, infections, inflammatory conditions or consumption of antibiotics during pregnancy [21,22]. Proposed dietary risk factors include also maternal prenatal and perinatal folate and iron status or polyunsaturated fatty acid (PUFA) intake [23,24,25].

Amongst the others, food restriction, difficult eating behaviors and GI disorders were easily recorded among medical conditions associated to ASDs. Indeed, children with ASD are very selective eaters (“picky eaters”) and most of them show aversions to specific food colors, texture, smells or other foods’ characteristics [26,27]. This exert a direct adversely effect on diet quality, nutritional deficiency and, on gut microbiota composition. Most of ASD patients that have a co-occurrence of GI disorders could be influenced by particular dietary habits that may exacerbate ASD symptomatology [28,29,30,31]. Immune dysfunction and gastrointestinal (GI) inflammation are also common in individuals with ASD and contribute to severity of behaviors [28,32,33]. Many ASD children have also been shown to carry abnormalities in GI physiology, including: increased intestinal permeability [34,35], overall microbiota alterations [36,37,38,39,40], and gut infection with cresol-producing Clostridium difficile [39,41,42,43,44].Recent evidences in human gut microbiota studies highlighted the existence of a close connection between gut and brain functions, the so called “gut-brain axis”, including neural, hormonal, immune, and metabolic pathways [45]. Neuroimmune pathways can contribute to ASD symptomatology via the gut–brain axis [46]. It has been proposed that cytokines associated with ASD, due to an inflamed gastrointestinal tract, may cross the blood-brain barrier and help an immune response in the brain, thus influencing behavior [46]. In this review we will highlight the emerging data about the relationship between gut microbiome, diet, GI symptoms and autism, and we will discuss nutritional criteria as intervention and strategy to ameliorate ASD symptoms.

## 2. Materials and Methods

### 2.1. Search Strategy

We conducted the review of literature to evaluate the altered gut microbiota and the effect of nutritional intervention in ASD patients. The research was conducted on PubMed, since 1955 to 2019 and using the following terms: “autism” or “autism spectrum disorder” or “diet” or “nutritional status” or “microbiota” or “microbiome” or “metabolites” or “dysbiosis” and “gastrointestinal symptoms”. All articles providing sufficient information about the relationship between the gut microbiota, nutritional intervention and ASD were included.

### 2.2. Selection Criteria

The inclusion criteria for study were as follows: (1) observational prospective and retrospective studies, case–control studies, cohort studies or systemic review; (2) investigating gut microbiota profiles and their metabolites in ASD children; (3) studies including information about nutritional intervention e nutritional status on ASD patients; and (4) studies written in English. All the studies that did not fall in the following criteria were excluded from the reviewing process.

## 3. The role of Nutrition and Interventions in ASD

### 3.1. Food Selectivity and ASD

Neurotypical children, especially preschoolers, are often referred to as “picky eater” and often show an attitude of preference towards certain foods and rejection of others. This alimentary conduct usually falls around the age of six and can be part of an adequate developmental framework typical of the developmental age [47,48].

In children with ASD, this picture is intensified, begins in a very early age and results in a real food selectivity framework. In addition, food problems tend to remain stable over time, with negative consequences on health and nutritional status. From a nutritional point of view this leads to an inadequate caloric intake and hence to nutritional deficits [49,50]. The importance of food regulation in children with ASD is emphasized in DSM-5, although it is not a diagnostic criterion [1]. 

**Figure 1:**
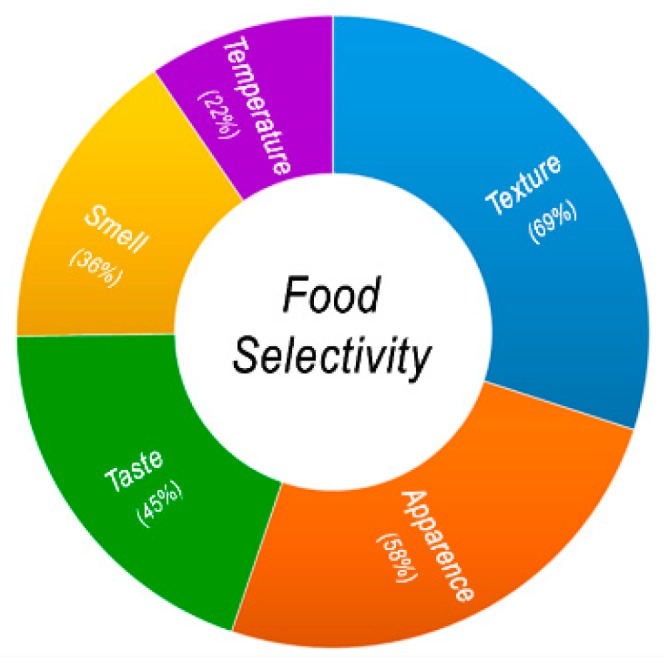
Factors that could determine food selectivity (data taken from the studies of Williams, Schreck and Klein [52,53,54]).

However, one of the major issues concerns the definition of selectivity, which complicates the evaluation and the comparison of the results of different studies. Atypical eating behaviors and the peculiar lifestyle of ASD (i.e., different levels of physical activity; idiosyncratic social skills; poor social interaction) are factors that imply risks of malnutrition, both in excess and in default [51]. Furthermore, studies have indicated that food selectivity is being determined by the following factors: texture (69%), appearance (58%), taste (45%), smell (36%), and temperature (22%), as well as reluctance to try new foods (69%) and a small repertoire of accepted foods (60%) (Figure 1) [52,53,54,55]. A strong preference for starches, snacks and processed foods, along with a rejection of fruits, vegetables or protein, is particularly common [56,57]. Increased consumption of snack foods and calorie-dense foods can lead to excessive weight gain, with related higher rates of obesity in ASD children than in unaffected children [58]. Indeed, obesity-related complications (e.g., hypertension, diabetes) are generally more prevalent among adults with ASD [59]. Nadon et al. found that nearly 90% of preschool and school age ASD children do not process sensory information, in particular related to touch, smell, sight, and hearing, in the same way as their typically developing peers [60]. Some studies reported that ASD children had strong food preferences [61].

Other factors related to food selectivity are linked to the way the food is presented (48.6%), to the use of certain utensils and to the characteristics of the tableware (13.8%) [53,62].

The study of Spek et al. [63] examined eating problems in the context of the Swedish Eating Assessment for Autism spectrum disorders [SWEAA] [64]. It has been shown that males with ASD can’t adapt their eating behavior to other people present and have problems doing two things simultaneously during a meal. Besides these, women with ASD showed eating rituals, a pronounced sensory sensitivity to the smell, taste, texture and visual appearance of food and were uncomfortable in sharing meals with other people [63].

Indeed, there are studies on the identification of specific sensorial patterns in ASD focused mainly on visual and auditory perception. A study on sensory profiles highlighted the existence of different clusters of sensory expression in ASD people [65]. In particular, the study identified a subpopulation of subjects among ASDs with particular taste/smell sensitive clusters, which could represent one of the major aspect in hampering feeding and introducing new foods. In another study, Miller identified three different cluster of modulating sensory sensitivity in ASDs and found a positive correlation between sensory overresponsivity (SOR) in ASDs and the severity level of food selectivity, expressed by number of foods accepted by the child [66].

Overall, the available evidence suggests that this food selectivity and an altered elaboration of sensory *stimuli* could imply a higher risk of nutritional deficiencies that could, in turn, affect gastrointestinal symptom and microbiota.

### 3.2. Nutrient Intake and ASDs.

**Figure 2:**
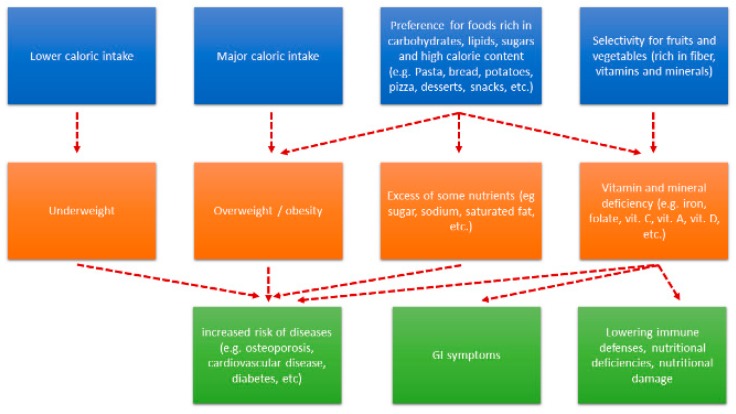
Synopsis of ASD food selectivity on nutritional status, anthropometric features and clinical conditions.

The ASD has been included among the psychiatric conditions associated with nutritional deficiencies due to food selectivity [67,68,69]. However, literature still shows conflicting results regarding the risk of nutritional deficits in children with ASD [70], especially because ASDs are compared with NTs. However, in many studies children with ASDs show a considerably smaller variety of foods, but authors report no overall differences in their total calories, carbohydrates, or fat intakes [62,71,72,73,74], suggesting that their satiety mechanisms are not impaired. Protein intake was adequate or quite similar to that of typically developing children [69,72,73,74,75,76,77]. Children with ASDs eat fewer vegetables and eat more energy-dense foods [76,78], so fiber intake was inadequate in a considerable number of children with ASDs [71,73,79,80]. Substantial number of subjects with ASDs had inadequate intakes of micronutrients. In particularly they showed deficiencies of few minerals such as calcium [67,69,71,73,75,77,79], iron [73,77], zinc [75,77,80], potassium [81], copper [81] and vitamins as vitamin A [71,75,77], vitamin D [67,69,73,78], vitamin E [71,73], riboflavin [77], vitamin C [75,78], vitamin B-12 [69,77,82], folic acid [75,82], and choline [80,83]. Excessive consumption of sodium was reported [79,84], probably due to the consumption of packaged foods. Some studies reported decreased bone development lower mineral density and a greater risk of fractures in children with ASD compared to controls (TDs), linked to a lack of calcium and vitamin D in the diet, despite good anthropometric growth [85,86,87,88]. Very interesting is the case of beta carotene excess reported in a case report of a 4-year-old ASD child with selective feeding and excessive consumption of carrot juice (>2.5 L/day) [89]. Cases of vitamin C deficiency with scurvy have been described in the literature [90,91,92,93,94,95]. However, dietary data obtained in the studies may be inaccurate due to the influence of parents, who, being concerned about the nutritional behavior of their children, do not actually reflect the correct nutritional approaches of their children. A schematic overview of food selectivity in ASDs’ children is provided below (Figure 2).

Therefore food selectivity and an inadequate nutrient intake could increase the risk of malnutrition in ASDs that ultimately leads to either obesity or undernutrition. In fact, it has been shown that this two conditions are associated with an altered composition and diversity of the gut microbiota compared to healthy individuals [96,97,98]. Furthermore this changes has been associated with altered SCFA composition, energy homeostasis, and inflammation [99]. Therefore it is important to take into account this influence that has the nutritional status on the intestinal microbiota, in order to choose the best nutritional approach for patients with ASD.

### 3.3. Effects of Dietary Interventions in ASD

Effects of dietary interventions in ASD have recently begun to emerge. It is important to understand what physiological effects dietary interventions may have, because individuals with ASD already exhibit difficult and picky eating behaviors [26,27]. So it is most important to investigate diets, because they could also aggravate the imbalances in gut microbiota composition and the GI problems. In the literature the most studied nutritional approaches are gluten-free/casein-free diet (GF/CFD), ketogenic diet (KD), the specific carbohydrate diet (SCD), and the Mediterranean diet (MD).

#### 3.3.1. Gluten-Free/Casein-Free Diet (GF/CFD)

One of the dietary interventions in ASD is the GF/CFD. This diet is characterized by exclusion of all food items containing wheat, oats, barley or rye, which are, all flours, bread, rusks, pasta, pastries, and other bakery products made with these cereals, while the elimination of casein means no intake of dairy products: milk, including breast milk, yogurt, cheese, butter, cream or ice cream, among others.

Evidences on healthy subjects showed that gluten-free diet has been associated with the reduction of beneficial gut bacteria populations, the increase in opportunistic pathogens and with immune-suppressive effects [100,101,102]. In the ASD population, and beyond, this diet could be recommended when an allergic or allergic intolerance is diagnosed [103,104]. Conflicting results have been recorded on ASD cohorts. Indeed, some evidence supports the use of this diet in the amelioration of ASD symptoms showing that the GF/CF diet decreases urine peptides, improves behavior [105] and decreases GI symptoms [106]; while other studies highlighted that the adoption of this elimination diet could decrease fiber intake [103], thus probably aggravating the GI problems.

To date evidence to support or refute GF/CF in ASD is limited and inadequate in terms of quantity, quality and multiple methodological limitations of studies in the literature.

#### 3.3.2. Ketogenic Diet (KD)

The KD is a high fat and low-carbohydrate diet and it is an effective treatment for epileptic patients that fails in responding to anticonvulsant medications [84]. KD has been researched in a variety of neurological conditions and also it has been suggested as a treatment for ASD. The administration of KD to individuals with ASD underlined positive effects especially for mild and moderate cases and some reports of improvements in seizure symptoms and behavioral deficits [84,107]. Biological findings for the effects of the KD come from animal studies. KD was shown to improve behavioral ASD deficits (such as sociability, repetitive behaviors, and social communication) in BTBR T^+^Itpr3^tf^/J mouse model of ASD (BTBR) [108]. KD treatment of BTBR mice also improved deficits typical of ASD related to myelin formation and white matter development/connectivity, acting on neurotransmitter signaling pathways including glutamate, serotonin, neuronal nitric acid synthase, and dopamine [109]. Additionally BTBR mice was characterized by a gut microbiota profile that was different from that of controls [110]. However, KD is associated with higher inflammatory and defective mitochondria risk and its side effects of constipation and reflux may worsen GI comorbidities in ASD. In a systematic review of KD in ASD it was concluded that the limited number of reports of improvements after treatment with the diet is not sufficient to attest to the practicability of KD as a treatment for the disorder [84].

#### 3.3.3. The Specific Carbohydrate Diet (SCD)

Another dietary protocol used in ASDs is the specific carbohydrate diet (SCD) but the studies conducted on this protocol are few. The SCD was developed in the 1930s as a dietary protocol intended for patients with celiac disease [111] but it is also employed to treat Crohn’s disease, ulcerative colitis, diverticulitis and chronic diarrhea [112,113,114]. Its purpose was to alleviate symptoms of malabsorption and to prevent growth of pathogenic gut microbiota. The diet recommends monosaccharides whose sources are fruit, some vegetables and honey, whereas the consumption of complex carbohydrates are restricted because they take much longer to digest than monosaccharides and may lead to difficulties of absorption and residual food becomes a breeding ground for pathogenic bacteria. We found one study that examined the implementation of an SCD protocol in a child with ASD, which showed that the SCD protocol was well tolerated in this 4 year old child diagnosed with ASD and fragile X syndrome (FXS), leading to improvements in growth status, gastrointestinal symptoms, and behaviors [112]. Further research is needed to further evaluate implementation of the SCD protocol in young children with ASD and/or FXS and GI concerns.

#### 3.3.4. Mediterranean Diet (MD)

The Mediterranean diet is characterized by a high consumption of fruits, vegetables, legumes, nuts, cereals, and olive oil, a moderate high intake of fish, dairy products, and alcohol (which comes primarily from wine), and a low intake of saturated lipids, sweets, and red and processed meat [115]. It represents the dietary pattern consumed by the populations situated near the Mediterranean Sea, and several studies have shown that this diet has beneficial effects against cardiovascular [116,117,118], metabolic [119,120] and mental diseases [121,122]. Actually, at our knowledge, no studies have reported the effect of MD on ASD patients. The only studies founded in the scientific literature about the influence of MD on neurodevelopmental diseases are about Attention Deficit Hyperactivity Disorder (ADHD). Ríos-Hernández et al., investigated the effect of MD in 60 children and adolescents with newly diagnosed ADHD. This was the first study showing that low adherence to the MD is associated with odds of an ADHD diagnosis in children and adolescents. Among the habits that characterize a MD pattern, individuals with ADHD more often missed having a second serving of fruit daily and showed reduced intakes of vegetables, pasta, and rice almost every day when compared with controls. Moreover, subjects with ADHD ate at fast-food restaurants and skipped breakfast more often than controls. In addition, a high consumption of sugar and candy, cola beverages, and noncola soft drinks and a low consumption of fatty fish were also associated with a higher prevalence of ADHD diagnosis. Authors found a positive relationship between a lower adherence to the MD and ADHD diagnoses. The findings suggest that certain dietary habits may play a role in ADHD development, even though further work is required to investigate causality and to determine if dietary manipulation could reverse the symptoms of ADHD, taking into consideration all potential factors [123].

## 4. The role of GI Symptoms, Gut Microbiota and Gut-Brain Axis in ASD

### 4.1. GI symptoms in Children with Autism

Individuals with ASD often suffer from gastrointestinal (GI) symptoms [30,124]. Frequent reports of GI symptoms in children with ASD are beginning to be clarified by research efforts examining the issue. Although the connection between gastrointestinal problems and autism is still not resolved and the prevalence of gastrointestinal symptoms varies from 23 to 70% [31,125,126,127,128,129].

This demonstrates a high variability in prevalence of GI problems that may be due to several differences across studies including: variations in the criteria used to define a GI symptom; the number of different GI symptoms considered; the definition of any particular GI symptom or lack of variations in methodology such as data source (medical chart versus self-report) or time period for reporting (last few months, lifetime, etc.); and study population characteristics such as age and other criteria for participation [130].

In the literature, we have found in-depth studies on 140–170 children with ASD, of which 24–63% had a history of at least one GI symptom, including: diarrhea or unformed stools, constipation, bloating, and/or gastroesophageal reflux (GERD) [31,125,131,132].

Another study on 150 children (50 ASD, 50 controls, and 50 children with other developmental disabilities (DD)) found that 70% of children with ASD presented GI symptoms, compared to 28% of typically developing children and 42% of DD children [126].

However, a study conducted in 2009 on people with ASD followed longitudinally up to 18 years, did not report an increased risk of GI diseases of an inflammatory and/or malabsorption nature compared to the typical development controls; the only significant difference found was the higher incidence of food selectivity and constipation in ASD people [127].

Therefore, it is not clear which kind of relationship correlates GI disorders and food selectivity, in fact, the malaise associated with GI disorders could increase feeding difficulties. Few evidences have been collected, at the moment, to deeply understand if GI symptoms may affect picky attitudes or if are principally the ASD dietary habits to influence GI disorders. Indeed, it could hypothesize that the picky attitude in ASD behavior could be due to a protective attitude, which the child implements to avoid discomfort resulting from the diet [133]. Interestingly other studies have shown that people with verbal and intestinal problems show poor appetite and react by rejecting a wide range of foods, and find it difficult to communicate their discomfort [51,134].

Moreover, food selectivity can exacerbate or determine GI symptoms (e.g., constipation) due to a diet rich in carbohydrates and poor in fiber that do not promote intestinal transit and can lead to constipation [127].

The presence of GI disorders together with food selectivity could constitute a specific clinical phenotype [31,127,135], characterized by frequent problematic behaviors, such as anxiety, self-aggression, sleep problems, resulting from both conditions [136]. Indeed correlation between certain behavioral problems, such as anxiety and aggression, and the increase in GI disorders is now known [137]. Indeed, abdominal pain, constipation, and/or diarrhea likely to produce frustration and may contribute to the severity of the disorder, with decreased ability to concentrate on tasks, behavior problems, and possibly aggression and self-abuse, especially in children unable to communicate their discomfort [29]. GI disorders also result in a decreased ability to learn toilet training, leading to increased frustration for the child and their parents/caregivers.

However, at the moment, it is difficult to precisely decipher the physiological processes that link together food selectivity and GI problems. What is certain is that both conditions, food selectivity and gastrointestinal disorders require attention from the clinician. Further studies characterized by a more accurate methodology, both in the selection of the samples and in the development and use of more accurate diagnostic tools, could allow a more precise estimation of the prevalence of GI disorders in the ASD [138,139,140].

### 4.2. GI Disorders, Microbiota and Microbiota-Gut-Brain Axis Alteration in ASD

GI disorders such as intestinal pain, constipation and diarrhea are often associated with an altered composition of the gut microbiota [28,132,140,141,142].

In the literature it has been reported that ASD children have altered gut microbiota profiles compared with NT children, although in some studies, no significant difference has been reported [19,20] Several studies on ASD have showed changes in the composition of the microbiota, particularly in the relative abundance of the mains gut bacterial phyla [36,37,143,144]. Indeed, some studies have revealed significant reductions in the relative abundance of *Prevotella*, *Coprococcus*, *Enterococcus*, *Lactobacillus*, *Streptococcus*, *Lactococcus*, *Staphylococcus*, *Ruminococcus*, and *Bifidobacterium* species in children with ASD compared with healthy controls [36,128,143]. In the scientific literature, some studies highlight a higher abundance of *Clostridia* and *Desulfovibrio* bacteria and a lower ratio of Bacteroidetes to Firmicutes in ASD [37,39,143,145]. A significantly higher prevalence of *Sutterella* species in biopsies taken from the GI tract of ASD children with GI disturbances compared to controls with GI disturbances has been found [146]. Wang et al. also demonstrated elevated numbers of *Sutterella*, as well as *Ruminococcus torques*, in the feces of children with ASD as compared to community controls [147]. Others studies observed, in ASD children, high abundance of: *Akkermansia muciniphilia*, [36,143,148] *Desulfovibrio*, [37] and *Faecalibacterium prausnitzii* [36]. A recent study, conducted on a set of 40 people with ASD and a control group of 40 NT, confirmed a different bacterial composition of the GI tract, but also showed an altered fungal colonization, in particular, the genus *Candida* was identified as the most important distribution, with a representation up to 2 times greater than that of the control population [149]. Intestinal dysbiosis is often associated, in the ASD population, with an alteration of the barrier of the intestinal mucosa with consequent increase of the intestinal permeability to exogenous substances of alimentary or bacterial origin, in some cases even neurotoxic [35].

A possible mechanism could be that this condition would allow macromolecules coming from GI tract to pass into the blood stream and exert an important systemic action; in particular, this action would apply at the level of the Central Nervous System (CNS) [150]. Indeed, microbiota and their ligands are crucial in maintaining the cell–cell junctions critical to barrier integrity, with GI barrier defects seen with dysbiosis [144]. Moreover, a higher intestinal permeability allow the increase in circulating bacteria-derived lipopolysaccharide (LPS) which leads to an immunological and inflammatory response, with an augmented systemic pro-inflammatory cytokines [151]. High levels of cytokines (e.g., IL-1B, IL-6, IL-8, and IL-12p40) have been reported in ASD children associated with poor communication and impaired social communication [32,152]. In a study that analyzed autopsy and cerebrospinal fluid (CSF) of individuals with ASD, a neuroinflammatory response involving excess microglial activation and increased proinflammatory cytokine profiles as compared to non-ASD controls was found [153]. The role of microglia deficits in neurological development disorders in a mouse model has emerged [154]. Therefore, this leads to the hypothesis that the leaky gut may play an important role in some behavioral manifestations of ASD children.

Thus the existence of a close connection between the gut and brain was highlighted, and that cross-communication occurs regularly. In fact, the CNS control the gut microbiome composition through peptides, which are sent upon satiation and thus affect nutrient availability. Furthermore, the hypothalamic–pituitary–adrenal (HPA) axis releases cortisol, which regulates intestinal motility, integrity and hypersecretion of CRH is a crucial factor in depression and anxiety disorders [155]. In turn, the immune and neuronal pathways regulate the secretion of mucin from intestinal epithelial cells, which control microbial populations within the intestine. However, communication is bidirectional and the intestinal microbiota is able to control the activity of the CNS through neural, endocrine, immune, and metabolic mechanisms that could have a possible influence on behaviours typical of ASD patients. [156]. A further confirmation of the possible central regulation mechanism of the gut-brain axis comes from studies on animal models, where it has been observed that an alteration of autonomic nervous system activity, such as anxiety and stress, could play a key role in the pathogenesis of increased permeability of the intestinal epithelium, found in the ASD population [40,157,158]. For example, germ-free (GF) mice showed reduced anxiety-like behavior and no spatial memory, altered neurotransmitter levels in the brain, and altered hypothalamic–pituitary–adrenal (HPA) axis activity [159,160,161,162]. Particularly intriguing for ASD is the influence of gut microbiota on the development of social behavior [163,164]. Indeed, the gut microbiota is reported to modulate structural and functional changes in the amygdala, a critical brain area for social and fear-related behaviors, which are associated with a variety of neuropsychiatric disorders [165]. A study conducted on early adolescence in mice showed that the modification of the intestinal microbiota alters their behavior and significantly reduces the neurotrophic factor (BDNF), oxytocin and vasopressin expression in the adult brain [166]. A study demonstrated that treatment with microbial-produced short-chain fatty acids (SCFAs) could rescue microglial function impaired in GF animals [167]. Furthermore, the microbiota affects the circulating levels of other mediators and substances, such as melatonin, serotonin, histamine and acetylcholine [168,169], which are important for brain maturation [170]. We can assume that if the hypothesis of a connection between symptoms related to autism and gastrointestinal disorders was confirmed, the manipulation of the intestinal microbiota, with supplementation with probiotics and treatment with Fecal Microbiota Transplantation (FMT), could constitute a therapeutic approach for the symptoms of autism and the associated medical comorbidities [171].

### 4.3. Focus on Bacterial Metabolites and Gut-Brain Axis

As we discussed, it is known that certain bacteria are able to produce different essential neurotransmitters and specific neuromodulators. Indeed, several neurotransmitters such as gamma-aminobutyric acid (GABA), serotonin, catecholamines and acetylcholine are produced by bacteria, some of which are inhabitants of the human gut. Indeed, researchers report that *Lactobacillus spp*. and *Bifidobacterium spp*. produce GABA [169]; *Escherichia spp*., *Bacillus spp.* and *Saccharomyces spp.* produce noradrenalin; *Candida spp*., *Streptococcus spp*., *Escherichia spp*. and *Enterococcus spp*. produce serotonin; *Bacillus spp*. produce dopamine; and *Lactobacillus spp*. produce acetylcholine [172]. Neurotransmitters secreted from gut bacteria may induce cells to release molecules that have the ability to modulate neural signaling within the enteric nervous system and subsequently control brain function and behavior, trough the microbiome-gut-brain axis. Significant deviations in the bacterial metabolites present in faeces and urine of children with ASD were seen [173]. Two possible pathways we hypothesize may be principally involved which are reviewed below.

#### 4.3.1. Short-Chain Fatty Acids (SCFAs) and Gut-Microbial Metabolites

Short-chain fatty acids (SCFAs) as acetic acid (AA), propionic acid (PPA), and butyric acid (BA), are the fermatation end-products of non-digested carbohydrates in the colon and have been suggested to have various health benefits to the host related to weight control, lipid profiles, and colon health [174].

However, the accumulation of SCFAs, and specifically of propionate, has also been shown to have broad effects on the nervous system physiology, and it is associated to the pathogenesis of ASD [175,176]. In fact, higher levels of AA and PPA that is used as a preservative in the food industry and can also induce autistic-like behaviors in rodents have been reported in ASD children [177,178]. At the same time, lower levels of BA, that can positively modulates neurotransmitter gene expression and can rescue behavioural abnormalities in mouse model, have been reported in ASD [179]. Moreover, ASD patients seem to be characterized by both elevated levels of SCFA concentrations in stool and serum, and increased level of SCFA-producing bacteria (e.g., *Clostridia*, *Desulfovibrio*, and *Bacteroides*) [29,36,180]. Thereby, translocation through the blood–brain barrier by transporters or by passive diffusion could cause potential effects on the brain and lead to development of some ASD symptoms [181]. The precise mechanisms of how SCFAs alter behavior in ASD are unknown, but effects on mitochondrial function (e.g., Krebs cycle) or epigenetic alterations may be involved [182].

In addition to direct effects on the brain, propionate has been shown to modulate 5-hydroxytryptamine (5’-HT) secretion in the gut and deplete 5’-HT and dopamine levels in the brain, which could potentially contribute to the hyperserotonemia observed in children with ASD [182,183,184].

Another metabolite that we could considered is *p*-cresol and its co-metabolite *p*-cresyl sulfate, which are phenolic compounds that are produced by bacteria such as *C. difficile* and *Bifidobacterium* [185,186,187]. It has been demonstrated that an early exposure to *p*-cresol may contribute to the severity of behavioral symptoms and cognitive impairment in ASD [185].

Furthermore, ASD patients have high level of free amino acids (FAAs) [186], which are derived from hydrolysis of proteins and peptides, like glutamate that may be involved in the etiopathogenesis of neurodevelopmental disorders [187].

This picture shows how there is a bidirectional influence between microbiota and diet, through the production of metabolites, which can be characterized through metabolomics and can help to delineate new therapeutical strategies in autistic patients.

#### 4.3.2. Neurotransmitters

In the last few years a role of the serotonin pathway in ASD, especially in the gut-brain axis, is emerging in the literature. Although most serotonin, or 5′-HT, is produced in the GI tract and can also be metabolized directly by the gut microbiota, it modulates neurodevelopment and might be important in social function and repetitive behavior [188]. High levels of 5′HT may be caused by a gastrointestinal 5′HT hypersecretion, produced by the enterochromaffin cells in the gut and it is involved in functions such as motility and secretion [189]. Furthermore, a study show the role of the 5′-HT as the link for the gut-brain-axis in ASD [190]. However, hyposerotonemia and lower synthesis of 5′HT in the brain in ASD children has been reported [191].

Some bacterial species that are known to influence 5′-HT metabolism (e.g., *Clostridium spp, Lactobacillus spp*) were observed to be increased in stool samples from ASD children. In patients with ASD, altered function and metabolism of neurotransmitters, such as 5′-HT and catecholamines, and dysfunction of the serotonergic system have been reported to contribute to symptomatology [188,192,193,194,195,196]. 5′-HT is elevated in whole blood and platelets in approximately 30% of children with ASD, making it a potential candidate as a biomarker for ASD [193]. Interestingly, administration of *Bacteroides fragilis* normalized plasma levels of 5′-HT in an animal model of ASD [197,198].

These data indicate that the gut microbiota could be involved in higher 5′-HT production, thus identifying 5′-HT as a potential pathway through which the gut microbiota and brain communicate in ASD. In ASD, abnormal intestinal permeability could allow 5′-HT to translocate into the systemic circulation, leading to elevated levels of blood 5′-HT [34,35,127,193]. Increased 5′-HT production by some species of the gut microbiota in ASD could deplete peripheral tryptophan availability. This corresponds to data showing decreased capacity for 5′-HT synthesis in children with ASD as well as to reports showing a worsening in repetitive behaviors in individuals with ASD after tryptophan depletion [191,199].

Lastly, higher levels of 5′-HT in children with ASD can be linked to intestinal inflammation and play an important role in intestinal inflammatory responses [200], so there is a connection between enteric serotonin production and dysbiosis. On the other hand, dysbiosis can decrease the number of amino acids that are absorbed from the diet and reduce the availability of tryptophan [201], that is a precursor for a number of metabolites as serotonin, thus creating a vicious cycle. Indeed, a lower level of tryptophan may influence the synthesis of serotonin in the brain, playing a role on the mood and cognitive impairment which characterize ASD children [202]. Thus, it can be proposed that the intestinal inflammatory response in children with ASD, which is exacerbated by gut microbiota, can lead to a further increase in 5′-HT levels and, ultimately, to upstream behavioral effects on the brain.

## 5. Conclusions

**Figure 3:**
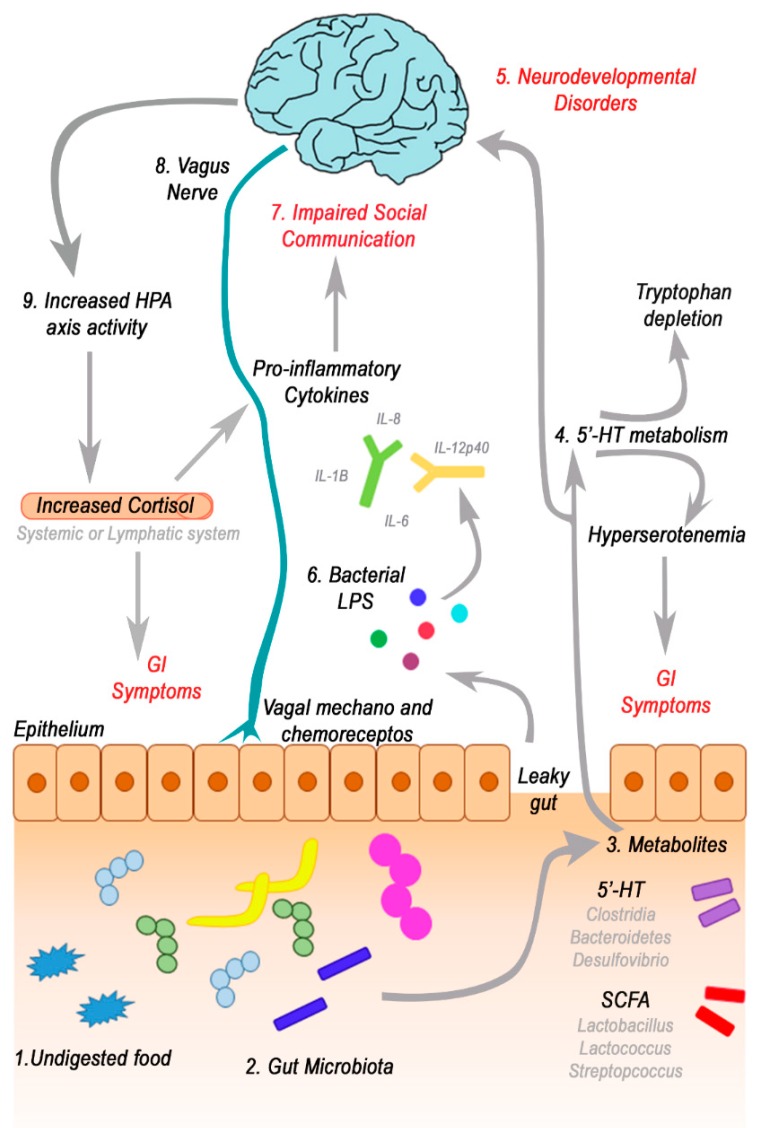
Role of the gut-brain axis in the etiology of ASD. (**1**,**2**) Food that escapes digestion can be used by the gut microbiota bacteria to produce metabolites (e.g., SCFAs and/or 5′-HT) that can be used by the host. Among metabolites (**3**) 5′-HT is produced particularly by *Lactobacillus*, *Streptococcus*, and *Lactococcus* species, while SCFAs (e.g., propionate) are produced by Clostridia, Bacteroidetes, and *Desulfovibrio* species. (**4**) Increased 5′-HT production by the microbiota acts on the metabolism of 5’-HT, leading to tryptophan depletion and contributing to hyperserotonemia, which is associated with GI Symptoms. (**5**) Intestinal permeability in children with ASD could allow passive diffusion of metabolites, and cause neurodevelopment disorders, such as behavioral and chemical changes (e.g., mood, cognitive state and emotion). (**6**,**7**) Moreover, higher intestinal permeability allow the increase in circulating bacteria-derived lipopolysaccharide (LPS), thus stimulating systemic pro-inflammatory cytokines production (e.g., IL-1B, IL-6, IL-8, and IL-12p40), which is associated with impaired social communication. (**8**) The vagal-mediated signaling from the gut microbiota to the brain can be transmitted through vagal chemoreceptors on mucosal villi that are activated by bacterial metabolites (e.g., 5′-HT, SCFAs) or by vagal mechanoreceptors that sense motility changes induced by bacterial species. (**9**) Gut microbiota influences the activity of Hypothalamus-Pituitary-Adrenal glands (HPA) axis that increased levels of cortisol in the systemic system. As a consequence, higher levels of cortisol may affect cytokines response and exacerbate GI symptoms.

It has been observed that ASD children are characterized by a strong food selectivity that consequently deeply influences their gut microbiota composition. Indeed, an increase in SCFA and 5′-HT-producing bacteria was observed in several studies on ASD patients. Increased levels of 5′-HT result in a different modulation of 5′-HT metabolism in the host, leading to tryptophan depletion and hyperserotoninemia, which may affect GI symptoms. Moreover, some ASDs are even characterized by higher levels of intestinal permeability which allow passive diffusion of bacteria-derived lipopolysaccharides (LPS) and metabolites through the intestinal barrier. As a consequence, an increase in pro-inflammatory cytokines (e.g., IL-1B, IL-6, IL-8, and IL-12p40) was observed, which are associated with impaired social communication and neurodevelopmental disorders. At the same time, gut-brain cross-talk through the vagus nerve and the hypothalamus-pituitary-adrenal (HPA) glands, influences vagal chemo- and mechanoreceptors on the mucosal villi and systemic cortisol levels, leading to an exacerbation of GI symptoms and inflammatory status (Figure 3). Further studies are needed to assess the effect of different dietary interventions (such as the Mediterranean diet) on GI symptoms and, as a consequence, how they may affect behavioral patterns associated to ASD conditions.

## References

[1] American Psychiatric Association (2013). Diagnostic and Statistical Manual of Mental Disorders.

[2] Munson J., Dawson G., Sterling L., Beauchaine T., Zhou A., Elizabeth K., Lord C., Rogers S., Sigman M., Estes A. (2008). Evidence for latent classes of IQ in young children with autism spectrum disorder. Am. J. Ment. Retard..

[3] Baio J., Wiggins L., Christensen D.L., Maenner M.J., Daniels J., Warren Z., Kurzius-Spencer M., Zahorodny W., Robinson Rosenberg C., White T. (2018). Prevalence of Autism Spectrum Disorder Among Children Aged 8 Years—Autism and Developmental Disabilities Monitoring Network, 11 Sites, United States, 2014. MMWR Surveill. Summ..

[4] Nomi J.S., Uddin L.Q. (2015). Developmental changes in large-scale network connectivity in autism. Neuroimage Clin..

[5] Uddin L.Q., Supekar K., Menon V. (2013). Reconceptualizing functional brain connectivity in autism from a developmental perspective. Front. Hum. Neurosci..

[6] Gaugler T., Klei L., Sanders S.J., Bodea C.A., Goldberg A.P., Lee A.B., Mahajan M., Manaa D., Pawitan Y., Reichert J. (2014). Most genetic risk for autism resides with common variation. Nat. Genet..

[7] Huguet G., Ey E., Bourgeron T. (2013). The Genetic Landscapes of Autism Spectrum Disorders. Annu. Rev. Genom. Hum. Genet..

[8] Sandin S., Lichtenstein P., Kuja-Halkola R., Hultman C., Larsson H., Reichenberg A. (2017). The Heritability of Autism Spectrum Disorder. JAMA.

[9] Chaste P., Leboyer M. (2012). Autism risk factors: Genes, environment, and gene-environment interactions. Dialogues Clin. Neurosci..

[10] (2013). Cross-Disorder Group of the Psychiatric Genomics Consortium Genetic relationship between five psychiatric disorders estimated from genome-wide SNPs. Nat. Genet..

[11] Durand C.M., Betancur C., Boeckers T.M., Bockmann J., Chaste P., Fauchereau F., Nygren G., Rastam M., Gillberg I.C., Anckarsäter H. (2007). Mutations in the gene encoding the synaptic scaffolding protein SHANK3 are associated with autism spectrum disorders. Nat. Genet..

[12] Griswold A.J., Ma D., Cukier H.N., Nations L.D., Schmidt M.A., Chung R.H., Jaworski J.M., Salyakina D., Konidari I., Whitehead P.L. (2012). Evaluation of copy number variations reveals novel candidate genes in autism spectrum disorder-associated pathways. Hum. Mol. Genet..

[13] Guilmatre A., Huguet G., Delorme R., Bourgeron T. (2014). The emerging role of *SHANK* genes in neuropsychiatric disorders: SHANK Genes in Neuropsychiatric Disorders. Dev. Neurobiol..

[14] Leblond C.S., Nava C., Polge A., Gauthier J., Huguet G., Lumbroso S., Giuliano F., Stordeur C., Depienne C., Mouzat K. (2014). Meta-analysis of SHANK Mutations in Autism Spectrum Disorders: A Gradient of Severity in Cognitive Impairments. PLoS Genet..

[15] Peça J., Feliciano C., Ting J.T., Wang W., Wells M.F., Venkatraman T.N., Lascola C.D., Fu Z., Feng G. (2011). Shank3 mutant mice display autistic-like behaviours and striatal dysfunction. Nature.

[16] Jonsson L., Zettergren A., Pettersson E., Hovey D., Anckarsäter H., Westberg L., Lichtenstein P., Lundström S., Melke J. (2014). Association study between autistic-like traits and polymorphisms in the autism candidate regions RELN, CNTNAP2, SHANK3, and CDH9/10. Mol. Autism.

[17] Poot M., Beyer V., Schwaab I., Damatova N., van’t Slot R., Prothero J., Holder S.E., Haaf T. (2010). Disruption of CNTNAP2 and additional structural genome changes in a boy with speech delay and autism spectrum disorder. Neurogenetics.

[18] Wilkinson B., Grepo N., Thompson B.L., Kim J., Wang K., Evgrafov O.V., Lu W., Knowles J.A., Campbell D.B. (2015). The autism-associated gene chromodomain helicase DNA-binding protein 8 (CHD8) regulates noncoding RNAs and autism-related genes. Transl. Psychiatry.

[19] Obrenovich M.E., Shamberger R.J., Lonsdale D. (2011). Altered heavy metals and transketolase found in autistic spectrum disorder. Biol. Trace Elem. Res..

[20] Splawski I., Yoo D.S., Stotz S.C., Cherry A., Clapham D.E., Keating M.T. (2006). CACNA1H mutations in autism spectrum disorders. J. Biol. Chem..

[21] Raz R., Roberts A.L., Lyall K., Hart J.E., Just A.C., Laden F., Weisskopf M.G. (2015). Autism spectrum disorder and particulate matter air pollution before, during, and after pregnancy: A nested case-control analysis within the Nurses’ Health Study II Cohort. Environ. Health Perspect..

[22] Herbert M.R. (2010). Contributions of the environment and environmentally vulnerable physiology to autism spectrum disorders. Curr. Opin. Neurol..

[23] Schmidt R.J., Lyall K., Hertz-Picciotto I. (2014). Environment and Autism: Current State of the Science. Cut Edge Psychiatry Pract..

[24] Lyall K., Munger K.L., O’Reilly É.J., Santangelo S.L., Ascherio A. (2013). Maternal Dietary Fat Intake in Association with Autism Spectrum Disorders. Am. J. Epidemiol..

[25] DeVilbiss E.A., Gardner R.M., Newschaffer C.J., Lee B.K. (2015). Maternal folate status as a risk factor for autism spectrum disorders: A review of existing evidence. Br. J. Nutr..

[26] Horvath K., Perman J.A. (2002). Autistic disorder and gastrointestinal disease. Curr. Opin. Pediatr..

[27] Cermak S.A., Curtin C., Bandini L.G. (2010). Food Selectivity and Sensory Sensitivity in Children with Autism Spectrum Disorders. J. Am. Diet. Assoc..

[28] McElhanon B.O., McCracken C., Karpen S., Sharp W.G. (2014). Gastrointestinal Symptoms in Autism Spectrum Disorder: A Meta-analysis. Pediatrics.

[29] Adams J.B., Johansen L.J., Powell L.D., Quig D., Rubin R.A. (2011). Gastrointestinal flora and gastrointestinal status in children with autism—Comparisons to typical children and correlation with autism severity. BMC Gastroenterol..

[30] Buie T., Campbell D.B., Fuchs G.J., Furuta G.T., Levy J., Vandewater J., Whitaker A.H., Atkins D., Bauman M.L., Beaudet A.L. (2010). Evaluation, diagnosis, and treatment of gastrointestinal disorders in individuals with ASDs: A consensus report. Pediatrics.

[31] Nikolov R.N., Bearss K.E., Lettinga J., Erickson C., Rodowski M., Aman M.G., McCracken J.T., McDougle C.J., Tierney E., Vitiello B. (2009). Gastrointestinal Symptoms in a Sample of Children with Pervasive Developmental Disorders. J. Autism Dev. Disord..

[32] Onore C., Careaga M., Ashwood P. (2012). The role of immune dysfunction in the pathophysiology of autism. BrainBehav. Immun..

[33] Mead J., Ashwood P. (2015). Evidence supporting an altered immune response in ASD. Immunol. Lett..

[34] D’Eufemia P., Celli M., Finocchiaro R., Pacifico L., Viozzi L., Zaccagnini M., Cardi E., Giardini O. (1996). Abnormal intestinal permeability in children with autism. Acta Paediatr..

[35] De Magistris L., Familiari V., Pascotto A., Sapone A., Frolli A., Iardino P., Carteni M., De Rosa M., Francavilla R., Riegler G. (2010). Alterations of the intestinal barrier in patients with autism spectrum disorders and in their first-degree relatives. J. Pediatr. Gastroenterol. Nutr..

[36] De Angelis M., Piccolo M., Vannini L., Siragusa S., De Giacomo A., Serrazzanetti D.I., Cristofori F., Guerzoni M.E., Gobbetti M., Francavilla R. (2013). Fecal Microbiota and Metabolome of Children with Autism and Pervasive Developmental Disorder Not Otherwise Specified. PLoS ONE.

[37] Finegold S.M., Dowd S.E., Gontcharova V., Liu C., Henley K.E., Wolcott R.D., Youn E., Summanen P.H., Granpeesheh D., Dixon D. (2010). Pyrosequencing study of fecal microflora of autistic and control children. Anaerobe.

[38] Mulle J.G., Sharp W.G., Cubells J.F. (2013). The Gut Microbiome: A New Frontier in Autism Research. Curr. Psychiatry Rep..

[39] Parracho H.M.R.T., Bingham M.O., Gibson G.R., McCartney A.L. (2005). Differences between the gut microflora of children with autistic spectrum disorders and that of healthy children. J. Med. Microbiol..

[40] Diaz Heijtz R., Wang S., Anuar F., Qian Y., Björkholm B., Samuelsson A., Hibberd M.L., Forssberg H., Pettersson S. (2011). Normal gut microbiota modulates brain development and behavior. Proc. Natl. Acad. Sci. USA.

[41] Elsden S.R., Hilton M.G., Waller J.M. (1976). The end products of the metabolism of aromatic amino acids by Clostridia. Arch. Microbiol..

[42] Finegold S.M., Molitoris D., Song Y., Liu C., Vaisanen M.L., Bolte E., McTeague M., Sandler R., Wexler H., Marlowe E.M. (2002). Gastrointestinal microflora studies in late-onset autism. Clin. Infect. Dis..

[43] Selmer T., Andrei P.I. (2001). p-Hydroxyphenylacetate decarboxylase from Clostridium difficile. A novel glycyl radical enzyme catalysing the formation of p-cresol. Eur. J. Biochem..

[44] Song Y.L., Liu C.X., McTeague M., Summanen P., Finegold S.M. (2004). Clostridium bartlettii sp. nov., isolated from human faeces. Anaerobe.

[45] Berding K., Donovan S.M. (2016). Microbiome and nutrition in autism spectrum disorder: Current knowledge and research needs. Nutr. Rev..

[46] De Theije C.G.M., Wu J., da Silva S.L., Kamphuis P.J., Garssen J., Korte S.M., Kraneveld A.D. (2011). Pathways underlying the gut-to-brain connection in autism spectrum disorders as future targets for disease management. Eur. J. Pharmacol..

[47] Carruth B.R., Ziegler P.J., Gordon A., Barr S.I. (2004). Prevalence of picky eaters among infants and toddlers and their caregivers’ decisions about offering a new food. J. Am. Diet. Assoc..

[48] Carruth B.R., Skinner J.D. (2000). Revisiting the picky eater phenomenon: Neophobic behaviors of young children. J. Am. Coll. Nutr..

[49] Bryant-Waugh R., Markham L., Kreipe R.E., Walsh B.T. (2010). Feeding and eating disorders in childhood. Int. J. Eat. Disord..

[50] Kreipe R.E., Palomaki A. (2012). Beyond picky eating: Avoidant/restrictive food intake disorder. Curr. Psychiatry Rep..

[51] Twachtman-Reilly J., Amaral S.C., Zebrowski P.P. (2008). Addressing feeding disorders in children on the autism spectrum in school-based settings: Physiological and behavioral issues. Lang. Speech Hear. Serv. Sch..

[52] Williams P.G., Dalrymple N., Neal J. (2000). Eating habits of children with autism. Pediatr. Nurs..

[53] Schreck K.A., Williams K. (2006). Food preferences and factors influencing food selectivity for children with autism spectrum disorders. Res. Dev. Disabil..

[54] Klein U., Nowak A.J. (1999). Characteristics of patients with autistic disorder (AD) presenting for dental treatment: A survey and chart review. Spec. Care Dentist..

[55] Marí-Bauset S., Zazpe I., Mari-Sanchis A., Llopis-González A., Morales-Suárez-Varela M. (2014). Food selectivity in autism spectrum disorders: A systematic review. J. Child Neurol..

[56] Sharp W.G., Berry R.C., McCracken C., Nuhu N.N., Marvel E., Saulnier C.A., Klin A., Jones W., Jaquess D.L. (2013). Feeding problems and nutrient intake in children with autism spectrum disorders: A meta-analysis and comprehensive review of the literature. J. Autism. Dev. Disord..

[57] Curtin C., Anderson S.E., Must A., Bandini L. (2010). The prevalence of obesity in children with autism: A secondary data analysis using nationally representative data from the National Survey of Children’s Health. BMC Pediatr..

[58] Berry R.C., Novak P., Withrow N., Schmidt B., Rarback S., Feucht S., Criado K.K., Sharp W.G. (2015). Nutrition Management of Gastrointestinal Symptoms in Children with Autism Spectrum Disorder: Guideline from an Expert Panel. J. Acad. Nutr. Diet..

[59] Cornish E. (1998). A balanced approach towards healthy eating in autism. J. Hum. Nutr. Diet..

[60] Nadon G., Feldman D.E., Dunn W., Gisel E. (2011). Mealtime problems in children with autism spectrum disorder and their typically developing siblings: A comparison study. Autism.

[61] Kerwin M.E., Eicher P.S., Gelsinger J. (2005). Parental Report of Eating Problems and Gastrointestinal Symptoms in Children with Pervasive Developmental Disorders. Child. Health Care.

[62] Schmitt L., Heiss C., Campbell E. (2008). A Comparison of Nutrient Intake and Eating Behaviors of Boys with and Without Autism. Top. Clin. Nutr..

[63] Spek A.A., van Rijnsoever W., van Laarhoven L., Kiep M. (2019). Eating Problems in Men and Women with an Autism Spectrum Disorder. J. Autism Dev. Disord..

[64] Karlsson L., Råstam M., Wentz E. (2013). The SWedish Eating Assessment for Autism spectrum disorders (SWEAA)-Validation of a self-report questionnaire targeting eating disturbances within the autism spectrum. Res. Dev. Disabil..

[65] Lane A.E., Molloy C.A., Bishop S.L. (2014). Classification of children with autism spectrum disorder by sensory subtype: A case for sensory-based phenotypes. Autism Res..

[66] Miller L.J., Anzalone M.E., Lane S.J., Cermak S.A., Osten E.T. (2007). Concept evolution in sensory integration: A proposed nosology for diagnosis. Am. J. Occup. Ther..

[67] Bandini L.G., Anderson S.E., Curtin C., Cermak S., Evans E.W., Scampini R., Maslin M., Must A. (2010). Food selectivity in children with autism spectrum disorders and typically developing children. J. Pediatr..

[68] Jen M., Yan A.C. (2010). Syndromes associated with nutritional deficiency and excess. Clin. Dermatol..

[69] Zimmer M.H., Hart L.C., Manning-Courtney P., Murray D.S., Bing N.M., Summer S. (2012). Food variety as a predictor of nutritional status among children with autism. J. Autism Dev. Disord..

[70] Marí-Bauset S., Llopis-González A., Zazpe-García I., Marí-Sanchis A., Morales-Suárez-Varela M. (2015). Nutritional status of children with autism spectrum disorders (ASDs): A case-control study. J. Autism Dev. Disord..

[71] Lockner D.W., Crowe T.K., Skipper B.J. (2008). Dietary intake and parents’ perception of mealtime behaviors in preschool-age children with autism spectrum disorder and in typically developing children. J. Am. Diet. Assoc..

[72] Emond A., Emmett P., Steer C., Golding J. (2010). Feeding symptoms, dietary patterns, and growth in young children with autism spectrum disorders. Pediatrics.

[73] Herndon A.C., DiGuiseppi C., Johnson S.L., Leiferman J., Reynolds A. (2009). Does nutritional intake differ between children with autism spectrum disorders and children with typical development?. J. Autism Dev. Disord..

[74] Levy S.E., Souders M.C., Ittenbach R.F., Giarelli E., Mulberg A.E., Pinto-Martin J.A. (2007). Relationship of dietary intake to gastrointestinal symptoms in children with autistic spectrum disorders. Biol. Psychiatry.

[75] Xia W., Zhou Y., Sun C., Wang J., Wu L. (2010). A preliminary study on nutritional status and intake in Chinese children with autism. Eur. J. Pediatr..

[76] Johnson C.R., Handen B.L., Mayer-Costa M., Sacco K. (2008). Eating habits and dietary status in young children with autism. J. Dev. Phys. Disabil..

[77] Cornish E. (2002). Gluten and casein free diets in autism: A study of the effects on food choice and nutrition. J. Hum. Nutr. Diet..

[78] Evans E.W., Must A., Anderson S.E., Curtin C., Scampini R., Maslin M., Bandini L. (2012). Dietary Patterns and Body Mass Index in Children with Autism and Typically Developing Children. Res. Autism Spectr. Disord..

[79] Bicer A.H., Alsaffar A.A. (2013). Body mass index, dietary intake and feeding problems of Turkish children with autism spectrum disorder (ASD). Res. Dev. Disabil..

[80] Hyman S.L., Stewart P.A., Schmidt B., Cain U., Lemcke N., Foley J.T., Peck R., Clemons T., Reynolds A., Johnson C. (2012). Nutrient intake from food in children with autism. Pediatrics.

[81] Malhi P., Venkatesh L., Bharti B., Singhi P. (2017). Feeding Problems and Nutrient Intake in Children with and without Autism: A Comparative Study. Indian J. Pediatr..

[82] Al-Farsi Y.M., Waly M.I., Deth R.C., Al-Sharbati M.M., Al-Shafaee M., Al-Farsi O., Al-Khaduri M.M., Gupta I., Ali A., Al-Khalili M. (2013). Low folate and vitamin B12 nourishment is common in Omani children with newly diagnosed autism. Nutrition.

[83] Hamlin J.C., Pauly M., Melnyk S., Pavliv O., Starrett W., Crook T.A., James S.J. (2013). Dietary intake and plasma levels of choline and betaine in children with autism spectrum disorders. Autism Res. Treat..

[84] Castro K., Faccioli L.S., Baronio D., Gottfried C., Perry I.S., dos Santos Riesgo R. (2015). Effect of a ketogenic diet on autism spectrum disorder: A systematic review. Res. Autism Spectr. Disord..

[85] Hediger M.L., England L.J., Molloy C.A., Yu K.F., Manning-Courtney P., Mills J.L. (2008). Reduced bone cortical thickness in boys with autism or autism spectrum disorder. J. Autism Dev. Disord..

[86] Neumeyer A.M., O’Rourke J.A., Massa A., Lee H., Lawson E.A., McDougle C.J., Misra M. (2015). Brief report: Bone fractures in children and adults with autism spectrum disorders. J. Autism Dev. Disord..

[87] Clark J.H., Rhoden D.K., Turner D.S. (1993). Symptomatic vitamin A and D deficiencies in an eight-year-old with autism. J. Parenter. Enter. Nutr..

[88] Stewart C., Latif A. (2008). Symptomatic nutritional rickets in a teenager with autistic spectrum disorder. Child Care Health Dev..

[89] Keown K., Bothwell J., Jain S. (2014). Nutritional implications of selective eating in a child with autism spectrum disorder. BMJ Case Rep..

[90] Duggan C.P., Westra S.J., Rosenberg A.E. (2007). Case records of the Massachusetts General Hospital. Case 23-2007. A 9-year-old boy with bone pain, rash, and gingival hypertrophy. N. Engl. J. Med..

[91] Gongidi P., Johnson C., Dinan D. (2013). Scurvy in an autistic child: MRI findings. Pediatr. Radiol..

[92] Duvall M.G., Pikman Y., Kantor D.B., Ariagno K., Summers L., Sectish T.C., Mullen M.P. (2013). Pulmonary hypertension associated with scurvy and vitamin deficiencies in an autistic child. Pediatrics.

[93] Kitcharoensakkul M., Schulz C.G., Kassel R., Khanna G., Liang S., Ngwube A., Baszis K.W., Hunstad D.A., White A.J. (2014). Scurvy revealed by difficulty walking: Three cases in young children. J. Clin. Rheumatol..

[94] Gulko E., Collins L.K., Murphy R.C., Thornhill B.A., Taragin B.H. (2015). MRI findings in pediatric patients with scurvy. Skelet. Radiol..

[95] Ma N.S., Thompson C., Weston S. (2016). Brief Report: Scurvy as a Manifestation of Food Selectivity in Children with Autism. J. Autism Dev. Disord..

[96] Turnbaugh P.J., Ley R.E., Mahowald M.A., Magrini V., Mardis E.R., Gordon J.I. (2006). An obesity-associated gut microbiome with increased capacity for energy harvest. Nature.

[97] Genton L., Cani P.D., Schrenzel J. (2015). Alterations of gut barrier and gut microbiota in food restriction, food deprivation and protein-energy wasting. Clin. Nutr..

[98] Tremaroli V., Kovatcheva-Datchary P., Bäckhed F. (2010). A role for the gut microbiota in energy harvesting?. Gut.

[99] De Clercq N.C., Groen A.K., Romijn J.A., Nieuwdorp M. (2016). Gut Microbiota in Obesity and Undernutrition. Adv. Nutr..

[100] Nistal E., Caminero A., Herrán A.R., Arias L., Vivas S., de Morales J.M.R., Calleja S., de Miera L.E.S., Arroyo P., Casqueiro J. (2012). Differences of small intestinal bacteria populations in adults and children with/without celiac disease: Effect of age, gluten diet, and disease. Inflamm. Bowel Dis..

[101] Di Cagno R., De Angelis M., De Pasquale I., Ndagijimana M., Vernocchi P., Ricciuti P., Gagliardi F., Laghi L., Crecchio C., Guerzoni M.E. (2011). Duodenal and faecal microbiota of celiac children: Molecular, phenotype and metabolome characterization. BMC Microbiol..

[102] De Palma G., Nadal I., Collado M.C., Sanz Y. (2009). Effects of a gluten-free diet on gut microbiota and immune function in healthy adult human subjects. Br. J. Nutr..

[103] Marí-Bauset S., Zazpe I., Mari-Sanchis A., Llopis-González A., Morales-Suárez-Varela M. (2014). Evidence of the gluten-free and casein-free diet in autism spectrum disorders: A systematic review. J. Child Neurol..

[104] Lange K.W., Hauser J., Reissmann A. (2015). Gluten-free and casein-free diets in the therapy of autism. Curr. Opin. Clin. Nutr. Metab. Care.

[105] Reichelt K.L., Knivsberg A.M. (2009). The possibility and probability of a gut-to-brain connection in autism. Ann. Clin. Psychiatry.

[106] Ghalichi F., Ghaemmaghami J., Malek A., Ostadrahimi A. (2016). Effect of gluten free diet on gastrointestinal and behavioral indices for children with autism spectrum disorders: A randomized clinical trial. World J. Pediatr..

[107] Evangeliou A., Vlachonikolis I., Mihailidou H., Spilioti M., Skarpalezou A., Makaronas N., Prokopiou A., Christodoulou P., Liapi-Adamidou G., Helidonis E. (2003). Application of a ketogenic diet in children with autistic behavior: Pilot study. J. Child Neurol..

[108] Ruskin D.N., Svedova J., Cote J.L., Sandau U., Rho J.M., Kawamura M., Boison D., Masino S.A. (2013). Ketogenic diet improves core symptoms of autism in BTBR mice. PLoS ONE.

[109] Mychasiuk R., Rho J.M. (2017). Genetic modifications associated with ketogenic diet treatment in the BTBRT+Tf/J mouse model of autism spectrum disorder. Autism Res..

[110] Newell C., Bomhof M.R., Reimer R.A., Hittel D.S., Rho J.M., Shearer J. (2016). Ketogenic diet modifies the gut microbiota in a murine model of autism spectrum disorder. Mol. Autism.

[111] Haas S.V., Haas M.P. (1955). The treatment of celiac disease with the specific carbohydrate diet; report on 191 additional cases. Am. J. Gastroenterol..

[112] Gottschall E. (2004). Digestion-gut-autism connection: The Specific Carbohydrate Diet. Med. Veritas J. Med. Truth.

[113] Suskind D.L., Wahbeh G., Gregory N., Vendettuoli H., Christie D. (2014). Nutritional therapy in pediatric Crohn disease: The specific carbohydrate diet. J. Pediatr. Gastroenterol. Nutr..

[114] Obih C., Wahbeh G., Lee D., Braly K., Giefer M., Shaffer M.L., Nielson H., Suskind D.L. (2016). Specific carbohydrate diet for pediatric inflammatory bowel disease in clinical practice within an academic IBD center. Nutrition.

[115] Štefan L., Prosoli R., Juranko D., Čule M., Milinović I., Novak D., Sporiš G. (2017). The Reliability of the Mediterranean Diet Quality Index (KIDMED) Questionnaire. Nutrients.

[116] Martinez-Gonzalez M.A., Bes-Rastrollo M. (2014). Dietary patterns, Mediterranean diet, and cardiovascular disease. Curr. Opin. Lipidol..

[117] De Lorgeril M., Salen P., Martin J.L., Monjaud I., Delaye J., Mamelle N. (1999). Mediterranean diet, traditional risk factors, and the rate of cardiovascular complications after myocardial infarction: Final report of the Lyon Diet Heart Study. Circulation.

[118] Ros E., Martínez-González M.A., Estruch R., Salas-Salvadó J., Fitó M., Martínez J.A., Corella D. (2014). Mediterranean diet and cardiovascular health: Teachings of the PREDIMED study. Adv. Nutr..

[119] Giugliano D., Esposito K. (2008). Mediterranean diet and metabolic diseases. Curr. Opin. Lipidol..

[120] Kesse-Guyot E., Ahluwalia N., Lassale C., Hercberg S., Fezeu L., Lairon D. (2013). Adherence to Mediterranean diet reduces the risk of metabolic syndrome: A 6-year prospective study. Nutr. Metab. Cardiovasc. Dis..

[121] Psaltopoulou T., Sergentanis T.N., Panagiotakos D.B., Sergentanis I.N., Kosti R., Scarmeas N. (2013). Mediterranean diet, stroke, cognitive impairment, and depression: A meta-analysis. Ann. Neurol..

[122] Muñoz M.A., Fíto M., Marrugat J., Covas M.I., Schröder H. (2009). REGICOR and HERMES investigators Adherence to the Mediterranean diet is associated with better mental and physical health. Br. J. Nutr..

[123] Ríos-Hernández A., Alda J.A., Farran-Codina A., Ferreira-García E., Izquierdo-Pulido M. (2017). The Mediterranean Diet and ADHD in Children and Adolescents. Pediatrics.

[124] Buie T., Fuchs G.J., Furuta G.T., Kooros K., Levy J., Lewis J.D., Wershil B.K., Winter H. (2010). Recommendations for evaluation and treatment of common gastrointestinal problems in children with ASDs. Pediatrics.

[125] Molloy C.A., Manning-Courtney P. (2003). Prevalence of chronic gastrointestinal symptoms in children with autism and autistic spectrum disorders. Autism.

[126] Valicenti-McDermott M., McVicar K., Rapin I., Wershil B.K., Cohen H., Shinnar S. (2006). Frequency of gastrointestinal symptoms in children with autistic spectrum disorders and association with family history of autoimmune disease. J. Dev. Behav. Pediatr..

[127] Ibrahim S.H., Voigt R.G., Katusic S.K., Weaver A.L., Barbaresi W.J. (2009). Incidence of gastrointestinal symptoms in children with autism: A population-based study. Pediatrics.

[128] Wang L.W., Tancredi D.J., Thomas D.W. (2011). The prevalence of gastrointestinal problems in children across the United States with autism spectrum disorders from families with multiple affected members. J. Dev. Behav. Pediatr..

[129] Gorrindo P., Williams K.C., Lee E.B., Walker L.S., McGrew S.G., Levitt P. (2012). Gastrointestinal dysfunction in autism: Parental report, clinical evaluation, and associated factors. Autism Res..

[130] Chaidez V., Hansen R.L., Hertz-Picciotto I. (2014). Gastrointestinal problems in children with autism, developmental delays or typical development. J. Autism Dev. Disord..

[131] Ming X., Brimacombe M., Chaaban J., Zimmerman-Bier B., Wagner G.C. (2008). Autism spectrum disorders: Concurrent clinical disorders. J. Child Neurol..

[132] Adams J.B., Holloway C.E., George F., Quig D. (2006). Analyses of toxic metals and essential minerals in the hair of Arizona children with autism and associated conditions, and their mothers. Biol. Trace Elem. Res..

[133] Horvath K., Papadimitriou J.C., Rabsztyn A., Drachenberg C., Tildon J.T. (1999). Gastrointestinal abnormalities in children with autistic disorder. J. Pediatr..

[134] Field D., Garland M., Williams K. (2003). Correlates of specific childhood feeding problems. J. Paediatr. Child Health.

[135] Whitehouse A.J.O., Maybery M., Wray J.A., Hickey M. (2011). No association between early gastrointestinal problems and autistic-like traits in the general population. Dev. Med. Child. Neurol..

[136] Prosperi M., Santocchi E., Balboni G., Narzisi A., Bozza M., Fulceri F., Apicella F., Igliozzi R., Cosenza A., Tancredi R. (2017). Behavioral Phenotype of ASD Preschoolers with Gastrointestinal Symptoms or Food Selectivity. J. Autism Dev. Disord..

[137] Fulceri F., Morelli M., Santocchi E., Cena H., Del Bianco T., Narzisi A., Calderoni S., Muratori F. (2016). Gastrointestinal symptoms and behavioral problems in preschoolers with Autism Spectrum Disorder. Dig. Liver Dis..

[138] Kuddo T., Nelson K.B. (2003). How common are gastrointestinal disorders in children with autism?. Curr. Opin. Pediatr..

[139] Holingue C., Newill C., Lee L.C., Pasricha P.J., Daniele Fallin M. (2018). Gastrointestinal symptoms in autism spectrum disorder: A review of the literature on ascertainment and prevalence. Autism Res..

[140] Adams J.B., Romdalvik J., Ramanujam V.M.S., Legator M.S. (2007). Mercury, lead, and zinc in baby teeth of children with autism versus controls. J. Toxicol. Environ. Health Part A.

[141] Konstantareas M.M., Homatidis S. (1987). Ear infections in autistic and normal children. J. Autism Dev. Disord..

[142] Niehus R., Lord C. (2006). Early medical history of children with autism spectrum disorders. J. Dev. Behav. Pediatr..

[143] Kang D.W., Park J.G., Ilhan Z.E., Wallstrom G., Labaer J., Adams J.B., Krajmalnik-Brown R. (2013). Reduced incidence of Prevotella and other fermenters in intestinal microflora of autistic children. PLoS ONE.

[144] Hsiao E.Y., McBride S.W., Hsien S., Sharon G., Hyde E.R., McCue T., Codelli J.A., Chow J., Reisman S.E., Petrosino J.F. (2013). Microbiota modulate behavioral and physiological abnormalities associated with neurodevelopmental disorders. Cell.

[145] Tomova A., Husarova V., Lakatosova S., Bakos J., Vlkova B., Babinska K., Ostatnikova D. (2015). Gastrointestinal microbiota in children with autism in Slovakia. Physiol. Behav..

[146] Williams B.L., Hornig M., Buie T., Bauman M.L., Cho Paik M., Wick I., Bennett A., Jabado O., Hirschberg D.L., Lipkin W.I. (2011). Impaired carbohydrate digestion and transport and mucosal dysbiosis in the intestines of children with autism and gastrointestinal disturbances. PLoS ONE.

[147] Wang L., Christophersen C.T., Sorich M.J., Gerber J.P., Angley M.T., Conlon M.A. (2013). Increased abundance of *Sutterella spp.* and *Ruminococcus torques* in feces of children with autism spectrum disorder. Mol. Autism.

[148] Xu M., Xu X., Li J., Li F. (2019). Association Between Gut Microbiota and Autism Spectrum Disorder: A Systematic Review and Meta-Analysis. Front. Psychiatry.

[149] Strati F., Cavalieri D., Albanese D., De Felice C., Donati C., Hayek J., Jousson O., Leoncini S., Renzi D., Calabrò A. (2017). New evidences on the altered gut microbiota in autism spectrum disorders. Microbiome.

[150] Julio-Pieper M., Bravo J.A., Aliaga E., Gotteland M. (2014). Review article: Intestinal barrier dysfunction and central nervous system disorders—A controversial association. Aliment. Pharmacol. Ther..

[151] Qin L., Wu X., Block M.L., Liu Y., Breese G.R., Hong J.S., Knapp D.J., Crews F.T. (2007). Systemic LPS causes chronic neuroinflammation and progressive neurodegeneration. Glia.

[152] Ashwood P., Krakowiak P., Hertz-Picciotto I., Hansen R., Pessah I., Van de Water J. (2011). Elevated plasma cytokines in autism spectrum disorders provide evidence of immune dysfunction and are associated with impaired behavioral outcome. Brain Behav. Immun..

[153] Vargas D.L., Nascimbene C., Krishnan C., Zimmerman A.W., Pardo C.A. (2005). Neuroglial activation and neuroinflammation in the brain of patients with autism. Ann. Neurol..

[154] Zhan Y., Paolicelli R.C., Sforazzini F., Weinhard L., Bolasco G., Pagani F., Vyssotski A.L., Bifone A., Gozzi A., Ragozzino D. (2014). Deficient neuron-microglia signaling results in impaired functional brain connectivity and social behavior. Nat. Neurosci..

[155] Trevarthen C., Aitken K., Nagy E., Delafield-Butt J., Vandekerckhove M. (2006). Collaborative Regulations of Vitality in Early Childhood: Stress in Intimate Relationships and Postnatal Psychopathology. Developmental Psychopathology.

[156] Wang Y., Kasper L.H. (2014). The role of microbiome in central nervous system disorders. Brain Behav. Immun..

[157] Keita A.V., Söderholm J.D. (2010). The intestinal barrier and its regulation by neuroimmune factors. Neurogastroenterol. Motil..

[158] Sudo N., Chida Y., Aiba Y., Sonoda J., Oyama N., Yu X.N., Kubo C., Koga Y. (2004). Postnatal microbial colonization programs the hypothalamic-pituitary-adrenal system for stress response in mice. J. Physiol..

[159] Neufeld K.M., Kang N., Bienenstock J., Foster J.A. (2011). Reduced anxiety-like behavior and central neurochemical change in germ-free mice. Neurogastroenterol. Motil..

[160] Gareau M.G., Wine E., Rodrigues D.M., Cho J.H., Whary M.T., Philpott D.J., Macqueen G., Sherman P.M. (2011). Bacterial infection causes stress-induced memory dysfunction in mice. Gut.

[161] Sampson T.R., Mazmanian S.K. (2015). Control of brain development, function, and behavior by the microbiome. Cell Host Microbe.

[162] Sudo N. (2012). Role of microbiome in regulating the HPA axis and its relevance to allergy. Chem. Immunol. Allergy.

[163] Desbonnet L., Clarke G., Shanahan F., Dinan T.G., Cryan J.F. (2014). Microbiota is essential for social development in the mouse. Mol. Psychiatry.

[164] Wong A.C.-N., Holmes A., Ponton F., Lihoreau M., Wilson K., Raubenheimer D., Simpson S.J. (2015). Behavioral Microbiomics: A Multi-Dimensional Approach to Microbial Influence on Behavior. Front. Microbiol..

[165] Stilling R.M., Ryan F.J., Hoban A.E., Shanahan F., Clarke G., Claesson M.J., Dinan T.G., Cryan J.F. (2015). Microbes & neurodevelopment—Absence of microbiota during early life increases activity-related transcriptional pathways in the amygdala. Brain Behav. Immun..

[166] Desbonnet L., Clarke G., Traplin A., O’Sullivan O., Crispie F., Moloney R.D., Cotter P.D., Dinan T.G., Cryan J.F. (2015). Gut microbiota depletion from early adolescence in mice: Implications for brain and behaviour. Brain Behav. Immun..

[167] Erny D., Hrabě de Angelis A.L., Jaitin D., Wieghofer P., Staszewski O., David E., Keren-Shaul H., Mahlakoiv T., Jakobshagen K., Buch T. (2015). Host microbiota constantly control maturation and function of microglia in the CNS. Nat. Neurosci..

[168] Forsythe P., Bienenstock J. (2010). Immunomodulation by commensal and probiotic bacteria. Immunol. Investig..

[169] Barrett E., Ross R.P., O’Toole P.W., Fitzgerald G.F., Stanton C. (2012). γ-Aminobutyric acid production by culturable bacteria from the human intestine. J. Appl. Microbiol..

[170] De Angelis M., Francavilla R., Piccolo M., De Giacomo A., Gobbetti M. (2015). Autism spectrum disorders and intestinal microbiota. Gut Microbes.

[171] Kang D.-W., Adams J.B., Coleman D.M., Pollard E.L., Maldonado J., McDonough-Means S., Caporaso J.G., Krajmalnik-Brown R. (2019). Long-term benefit of Microbiota Transfer Therapy on autism symptoms and gut microbiota. Sci. Rep..

[172] Dinan T.G., Cryan J.F. (2013). Melancholic microbes: A link between gut microbiota and depression?. Neurogastroenterol. Motil..

[173] Srikantha P., Mohajeri M.H. (2019). The Possible Role of the Microbiota-Gut-Brain-Axis in Autism Spectrum Disorder. Int. J. Mol. Sci..

[174] Byrne C.S., Chambers E.S., Morrison D.J., Frost G. (2015). The role of short chain fatty acids in appetite regulation and energy homeostasis. Int. J. Obes..

[175] Thomas R.H., Meeking M.M., Mepham J.R., Tichenoff L., Possmayer F., Liu S., MacFabe D.F. (2012). The enteric bacterial metabolite propionic acid alters brain and plasma phospholipid molecular species: Further development of a rodent model of autism spectrum disorders. J. Neuroinflamm..

[176] MacFabe D.F. (2015). Enteric short-chain fatty acids: Microbial messengers of metabolism, mitochondria, and mind: Implications in autism spectrum disorders. Microb. Ecol. Health Dis..

[177] Al-Lahham S.H., Peppelenbosch M.P., Roelofsen H., Vonk R.J., Venema K. (2010). Biological effects of propionic acid in humans; metabolism, potential applications and underlying mechanisms. Biochim. Biophys. Acta.

[178] Liu F., Li J., Wu F., Zheng H., Peng Q., Zhou H. (2019). Altered composition and function of intestinal microbiota in autism spectrum disorders: A systematic review. Transl. Psychiatry.

[179] Kratsman N., Getselter D., Elliott E. (2016). Sodium butyrate attenuates social behavior deficits and modifies the transcription of inhibitory/excitatory genes in the frontal cortex of an autism model. Neuropharmacology.

[180] Wang L., Christophersen C.T., Sorich M.J., Gerber J.P., Angley M.T., Conlon M.A. (2012). Elevated fecal short chain fatty acid and ammonia concentrations in children with autism spectrum disorder. Dig. Dis. Sci..

[181] Conn A.R., Fell D.I., Steele R.D. (1983). Characterization of alpha-keto acid transport across blood-brain barrier in rats. Am. J. Physiol..

[182] Reigstad C.S., Salmonson C.E., Rainey J.F., Szurszewski J.H., Linden D.R., Sonnenburg J.L., Farrugia G., Kashyap P.C. (2015). Gut microbes promote colonic serotonin production through an effect of short-chain fatty acids on enterochromaffin cells. FASEB J..

[183] El-Ansary A., Al-Ayadhi L. (2014). Relative abundance of short chain and polyunsaturated fatty acids in propionic acid-induced autistic features in rat pups as potential markers in autism. Lipids Health Dis..

[184] Al-Ghamdi M., Al-Ayadhi L., El-Ansary A. (2014). Selected biomarkers as predictive tools in testing efficacy of melatonin and coenzyme Q on propionic acid—Induced neurotoxicity in rodent model of autism. BMC Neurosci..

[185] Persico A.M., Napolioni V. (2013). Urinary p-cresol in autism spectrum disorder. Neurotoxicol. Teratol..

[186] Ming X., Stein T.P., Barnes V., Rhodes N., Guo L. (2012). Metabolic perturbance in autism spectrum disorders: A metabolomics study. J. Proteome Res..

[187] Shimmura C., Suda S., Tsuchiya K.J., Hashimoto K., Ohno K., Matsuzaki H., Iwata K., Matsumoto K., Wakuda T., Kameno Y. (2011). Alteration of plasma glutamate and glutamine levels in children with high-functioning autism. PLoS ONE.

[188] Muller C.L., Anacker A.M.J., Veenstra-VanderWeele J. (2016). The serotonin system in autism spectrum disorder: From biomarker to animal models. Neuroscience.

[189] Gheorghe C.E., Martin J.A., Manriquez F.V., Dinan T.G., Cryan J.F., Clarke G. (2019). Focus on the essentials: Tryptophan metabolism and the microbiome-gut-brain axis. Curr. Opin. Pharmacol..

[190] Israelyan N., Margolis K.G. (2018). Serotonin as a link between the gut-brain-microbiome axis in autism spectrum disorders. Pharmacol. Res..

[191] Chugani D.C., Muzik O., Behen M., Rothermel R., Janisse J.J., Lee J., Chugani H.T. (1999). Developmental changes in brain serotonin synthesis capacity in autistic and nonautistic children. Ann. Neurol..

[192] Melke J., Goubran Botros H., Chaste P., Betancur C., Nygren G., Anckarsäter H., Rastam M., Ståhlberg O., Gillberg I.C., Delorme R. (2008). Abnormal melatonin synthesis in autism spectrum disorders. Mol. Psychiatry.

[193] Gabriele S., Sacco R., Persico A.M. (2014). Blood serotonin levels in autism spectrum disorder: A systematic review and meta-analysis. Eur. Neuropsychopharmacol..

[194] Schain R.J., Freedman D.X. (1961). Studies on 5-hydroxyindole metabolism in autistic and other mentally retarded children. J. Pediatr..

[195] Anderson G.M., Freedman D.X., Cohen D.J., Volkmar F.R., Hoder E.L., McPhedran P., Minderaa R.B., Hansen C.R., Young J.G. (1987). Whole blood serotonin in autistic and normal subjects. J. Child Psychol. Psychiatry.

[196] Hanley H.G., Stahl S.M., Freedman D.X. (1977). Hyperserotonemia and amine metabolites in autistic and retarded children. Arch. Gen. Psychiatry.

[197] Yano J.M., Yu K., Donaldson G.P., Shastri G.G., Ann P., Ma L., Nagler C.R., Ismagilov R.F., Mazmanian S.K., Hsiao E.Y. (2015). Indigenous bacteria from the gut microbiota regulate host serotonin biosynthesis. Cell.

[198] Marler S., Ferguson B.J., Lee E.B., Peters B., Williams K.C., McDonnell E., Macklin E.A., Levitt P., Gillespie C.H., Anderson G.M. (2016). Brief Report: Whole Blood Serotonin Levels and Gastrointestinal Symptoms in Autism Spectrum Disorder. J. Autism Dev. Disord..

[199] McDougle C.J., Naylor S.T., Cohen D.J., Aghajanian G.K., Heninger G.R., Price L.H. (1996). Effects of tryptophan depletion in drug-free adults with autistic disorder. Arch. Gen. Psychiatry.

[200] Bischoff S.C., Mailer R., Pabst O., Weier G., Sedlik W., Li Z., Chen J.J., Murphy D.L., Gershon M.D. (2009). Role of serotonin in intestinal inflammation: Knockout of serotonin reuptake transporter exacerbates 2,4,6-trinitrobenzene sulfonic acid colitis in mice. Am. J. Physiol. Gastrointest. Liver Physiol..

[201] Kraneveld A.D., Szklany K., de Theije C.G.M., Garssen J. (2016). Gut-to-Brain Axis in Autism Spectrum Disorders: Central Role for the Microbiome. Int. Rev. Neurobiol..

[202] Fattorusso A., Di Genova L., Dell’Isola G.B., Mencaroni E., Esposito S. (2019). Autism Spectrum Disorders and the Gut Microbiota. Nutrients.

